# Undiagnosed Depression and Its Effects on Patients With Systemic Lupus Erythematosus

**DOI:** 10.7759/cureus.53064

**Published:** 2024-01-27

**Authors:** Zaid Tayyab, Haseeb Khan, Samina Saeed, Saba Saif, Sana Haseeb Khan, Muhammad Ijaz Bhatti

**Affiliations:** 1 Rheumatology, Fatima Memorial Hospital College of Medicine and Dentistry, Lahore, PAK; 2 Rheumatology, Dorset County Hospital, Dorchester, GBR; 3 Medicine, Allama Iqbal Medical College, Lahore, PAK; 4 Medicine, CMH (Combined Military Hospital) Lahore Medical College and Institute of Dentistry, Lahore, PAK; 5 Pathology and Laboratory Medicine, Al Aleem Medical College, Lahore, PAK; 6 Cardiology, Gulab Devi Hospital/Al Aleem Medical College, Lahore, PAK

**Keywords:** effects, prevalence, autoimmune, disease activity, systemic lupus erythematosus, undiagnosed depression

## Abstract

Introduction

Different organs and organ systems are affected by a well-known chronic immune disorder called systemic lupus erythematosus (SLE). Besides the physical harm caused by this disorder, it affects the mental health of patients in a greater ratio by causing depression and anxiety. The objective of this study is to assess the prevalence of undiagnosed depression and its effects on patients with systemic lupus erythematosus.

Material and methods

This prospective cross-sectional study was carried out in the Rheumatology outpatient department of Fatima Memorial Hospital (FMH), Lahore, from November 2022 to February 2023. All study subjects had been given a prior diagnosis of SLE based on the 2012 Systemic Lupus International Collaborating Clinics (SLICC) criteria. Three sections comprised the survey form: section I asked questions about socio-demographic information (gender and age); section II assessed the degree of mental illness activity; and section III assessed the degree of SLE disease activity. The nine-item PHQ-9 (Patient Health Questionnaire 9) scale, which is used to diagnose severe depressive disorder, was utilized to measure depression. To compare categorical variables, we applied Fisher's exact tests and chi-square; for continuous variables, we utilized the student's t-test. The statistical analysis was conducted using SPSS software for Windows Version 21.0 (IBM Corp., Armonk, NY, USA), with a significance threshold of p-value <0.05.

Results

Mild, moderate, and severe depression were present in 20%, 37.5%, and 37.5% of the patients, respectively. Pearson correlation of disease severity was strongly positive with depression (R^2^=0.634, p=0.01). The correlation was statistically significant.

Conclusion

Our research indicates that depression is a real problem for SLE patients. There is a positive correlation between the activity of the disease and the intensity of depression.

## Introduction

Different organs and organ systems are affected by a well-known chronic immune disorder called systemic lupus erythematosus (SLE). Besides the physical harm caused by this disorder, it affects the mental health of patients in a greater ratio by causing depression and anxiety. The incidence of anxiety and depression in SLE patients has been examined through various investigations; the findings range from 2.9% to 84.9% for anxiety and 2.1% to 78.6% for depression [1]. Although an extensive amount of research has been done, conventional clinical practice occasionally overlooks or delays diagnosing anxiety and depression in SLE patients. Additionally, many research studies have demonstrated a high correlation between depression, anxiety, and SLE. These correlations have been linked to suicidal ideas, an increase in functional limitations, and a reduction in treatment adherence [2,3]. Ultimately, the patients’ living standards are significantly reduced by these conditions [4-6].

Early detection of depression and anxiety is essential because there is a greater tendency and risk of mental illnesses in SLE patients. Many studies have been conducted to examine the impact of depression and anxiety in individuals with SLE, and the results have identified several variables associated with their higher prevalence. Research on disease activity among these factors has been done in-depth. Regretfully, there is still a debate over the exact nature of the connection between disease activity, depression, and anxiety. A correlation between elevated disease activity and greater susceptibility to depression and anxiety has been suggested by several studies. Conversely, other investigations have not discovered any connection between the occurrence of these signs and elevated disease activity. We conducted a cross-sectional, observational, descriptive, single-center study in SLE patients to investigate the relationship between depression and SLE disease activity to resolve this ongoing controversy.

## Materials and methods

This prospective cross-sectional study was carried out in the Rheumatology outpatient department, Fatima Memorial Hospital (FMH), Lahore, from November 2022 to February 2023, after approval (Ref. No. FMH-07/11/2022-IRB-1328). A total of 40 patients with a prior diagnosis of SLE based on the 2012 Systemic Lupus International Collaborating Clinics (SLICC criteria) were enrolled using convenience sampling. The requirements for participation included being at least 18 years old. The following conditions had to be met in order to be excluded: (1) a record of depression or anxiety before the SLE diagnosis; (2) previous mental health therapy; (3) background of alcohol or other drug abuse; (4) significant illnesses of the heart, liver, kidney, or other organs due to any other medical disease, (5) pregnancy, (6) maintenance dose of prednisolone > 7.5 mg per day or equivalent, and (7) treatment with biologics. The local ethics committee accepted the trial after receiving signed consent from all participants.

Three sections comprised the survey form: section I asked questions about sociodemographic information (gender and age); section II assessed the degree of mental illness activity; and section III assessed the degree of SLE disease activity.

With the help of the Systemic Lupus Erythematosus Disease Activity Index (SLEDAI) score, the disease activity was evaluated, with the readings varying from 0 to 5, with 0=no activity, 1-5=mild, 6-10=moderate, and >11=severe disease activity.

The nine-item PHQ-9 (Patient Health Questionnaire 9) scale, which is used to diagnose severe depressive disorder, was utilized to measure depression. Every item was evaluated using a four-point Likert scale (0-3), with scores falling between 0 and 27. More serious depression was indicated by higher scores: non-depression at >0 scores, mild depression at 5, moderate depression at 10, and severe depression at 15. It was demonstrated that the PHQ-9 possesses strong psychometric properties.

The data were displayed as mean and standard deviation for continuous variables and as counts and percentages for categorical variables. To compare categorical variables, we applied Fisher's exact tests and chi-square; for continuous variables, we utilized the student's t-test. The statistical analysis was conducted using SPSS software for Windows Version 21.0 (IBM Corp., Armonk, NY, USA), with a significance threshold of p-value<0.05.

## Results

**Figure 1:**
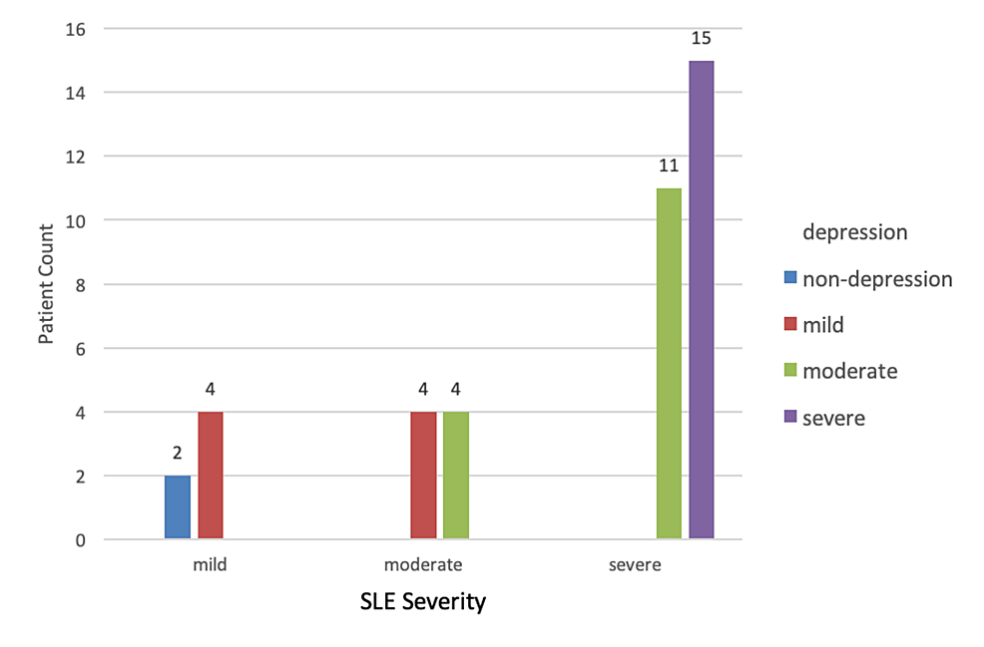
Depression in various disease-severity states

**Figure 2:**
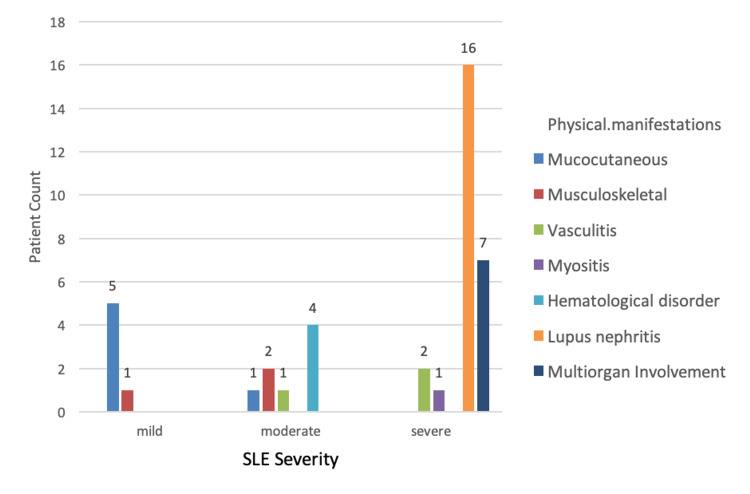
Physical manifestations in various SLE severity states SLE: systemic lupus erythematosus

**Figure 3:**
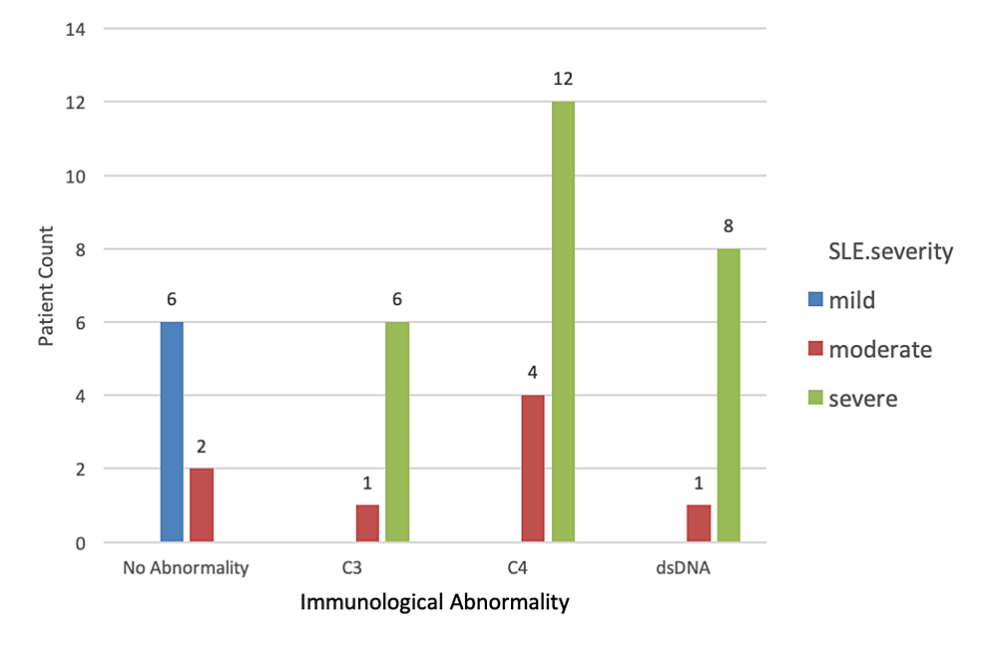
SLE severity with various immunological abnormalities SLE: systemic lupus erythematosus

A total of 40 patients were included in the study, out of which 92.5% (n=37) were females and 7.5% (n=3) were males with a female-to-male ratio of 37:3 (Table 1). The mean age of all the patients was 32.25±7.60 years. The mean age at diagnosis was 28 ± 4 years. The median duration of the disease was approximately three years with a minimum duration of one year to a maximum duration of eight years. All the patients were on treatment with hydroxychloroquine. Additionally, 40% (n=16) were taking mycophenolate mofetil, 37.5% (n=15) azathioprine, and 7.5 % (n=3) methotrexate. In addition to this, 72.5% of the patients (n=29) were on a maintenance dose of prednisolone. Immunological abnormalities on investigations were found in 80% (n=32) of the patients having moderate to severe disease (Figure 1). The majority of patients (65%; n=26) had severe disease, out of which 61.5% (n=16) had lupus nephritis, 26.9% (n=7) multiorgan involvement, 7.6 % (n=2) vasculitis, and 3.8% (n=1) myositis. Twenty percent (20%; n=8) had moderately active disease with predominant hematological involvement, seen in 20% (n=4) patients. Fifteen percent (15%; n=6) had mild disease with 83% (n=5) having mucocutaneous involvement (Figure 2). Mild, moderate, and severe depression was present in 20%, 37.5%, and 37.5% of the patients, respectively (Figure 3). The Pearson correlation of disease severity was strongly positive with depression (R2=0.634, p=0.01). The correlation was statistically significant (Table 2).

**Table 1:** Demographic data of the patients

Variable	Value (n=40)
Age (years)	32.25±7.60
Gender (male/female), N (%)	3 (7.5)/37 (92.5)

**Table 2:** Disease-related data of the patients SLEDAI: Systemic Lupus Erythematosus Disease Activity Index; SLE: systemic lupus erythematosus; PHQ-9: Patient Health Questionnaire 9

Variable	Value (n=40)
SLEDAI score	26.70±17.01
SLE severity, N (%)	
Mildly	6 (15)
Moderate	8 (20)
Severe	26 (65)
PHQ-9 score	12.45±4.48
Depression severity, N (%)	
Non-depression	2 (5)
Mild depression	8 (20)
Moderate depression	15 (37.5)
Severe depression	15 (37.5)
Pearson correlation (SLE, depression)	R^2^=0.634, p=0.01

## Discussion

In the past few years, it has been difficult to diagnose depression in SLE patients at an early stage, in part because of doctors' predisposition to focus on physical signs and in part because there is currently no trustworthy testing tool. Mok et al. examined the effect of depression on the standard of life in an investigation including SLE patients, highlighting the significance of determining the incidence of depression in making clinical decisions [7-9].

We examined depression in patients with SLE in our research; the high ratio made it evident that the timely detection of depression was essential. We also showed a direct correlation between illness activity and depression. According to the former research study, numerous characteristics, including specific organ involvement, Ab (antibodies), unemployment, exhaustion, sleep quality, age, cytokines, disease activity, glucocorticosteroid use, and many more, have been linked to anxiety and depression in lupus patients. These results imply that the intricate interactions between social, physiological, biological, environmental, and economic factors are most likely the cause of depression and anxiety in lupus [10-16].

Among the many characteristics associated with the condition, the connection between disease activity and depression in SLE has been well-investigated, but with varying outcomes. We showed in our study how the depression ratios varied across individuals at various phases of illness activity. Interestingly, the proportion of patients with depression was significantly higher in moderate-to-severe disease activity than in mild disease activity, suggesting that SLE disease activity plays a critical role in the intensity of depression and anxiety. This is in agreement with the results of Nery et al., who demonstrated a relationship between SLE disease activity and the severity of depression as determined by SLEDAI [17].

Research by Tay et al. and Mak et al. further indicates that increased SLE activity led to significant anxiety problems even though the participants adjusted to the signs of depression [18,19]. Conversely, some studies show no notable link between individuals who have high SELENA-SLEDAI scores and serious depressive disorders and those who do not have serious depression [13]. Furthermore, research by Jarpa et al. indicates that there is no connection between prevalent mental illnesses, in essence, depression and anxiety, and SLEDAI-evaluated SLE disease activity [20]. The reasons behind the discrepancies in research findings include the existence of several criteria for depressive illness, the use of various screening tools, and the use of distinct research approaches [21].

According to our research, the management of depression in addition to immune-suppressive medications should be considered if the PHQ-9 score indicates moderate-to-severe depression. Nevertheless, there were some limitations to our research, including small sample size, the possibility of questionnaire participation bias, the lack of longitudinal data, and the lack of a relationship between severe depression and signs and symptoms of depression. Admitted patients and those on higher steroid doses were excluded, which led to a further reduction in the sample size. Even though previous studies also had limits and inconsistent outcomes, our approach provides a foundation for further research in this particular area.

## Conclusions

SLE is a chronic multisystemic autoimmune disorder that can affect the mental health of patients to a greater extent. Unfortunately, treatment is focused on immunosuppressive medications and the psychological aspect is neglected, which can adversely affect the quality of life. Our research indicates that the majority of patients had undiagnosed depression and there was a positive correlation between disease activity and the intensity of depression. It is proposed that the treatment of depression is a vital component in the management of these patients and should be offered where appropriate.
